# Open Access Publishing in the Field of Medical Informatics

**DOI:** 10.1007/s10916-017-0729-0

**Published:** 2017-04-01

**Authors:** Stefanie Kuballa

**Affiliations:** Peter L. Reichertz Institute for Medical Informatics, University of Braunschweig – Institute of Technology and Hannover Medical School, Muehlenpfordtstr. 23, 38106 Braunschweig, Germany

**Keywords:** Open access, Medical informatics, Publishing, Charges, Licensing

## Abstract

The open access paradigm has become an important approach in today’s information and communication society. Funders and governments in different countries stipulate open access publications of funded research results. Medical informatics as part of the science, technology and medicine disciplines benefits from many research funds, such as National Institutes of Health in the US, Wellcome Trust in UK, German Research Foundation in Germany and many more. In this study an overview of the current open access programs and conditions of major journals in the field of medical informatics is presented. It was investigated whether there are suitable options and how they are shaped. Therefore all journals in Thomson Reuters Web of Science that were listed in the subject category “Medical Informatics” in 2014 were examined. An Internet research was conducted by investigating the journals’ websites. It was reviewed whether journals offer an open access option with a subsequent check of conditions as for example the type of open access, the fees and the licensing. As a result all journals in the field of medical informatics that had an impact factor in 2014 offer an open access option. A predominantly consistent pricing range was determined with an average fee of 2.248 € and a median fee of 2.207 €. The height of a journals’ open access fee did not correlate with the height of its Impact Factor. Hence, medical informatics journals have recognized the trend of open access publishing, though the vast majority of them are working with the hybrid method. Hybrid open access may however lead to problems in questions of double dipping and the often stipulated gold open access.

## Introduction

Existing publication models charge readers for accessing journal or book content of publishers. Within the framework of changes and advancements in the information and communication technologies this traditional publishing model is also experiencing change [1, 2]. The Internet allows publishing articles or whole journals online, so it has become common practice to offer content on publisher websites and e-print archives alongside the printed version [3]. The open access model is a further logical consequence of this development.

The free access to scientific journal articles is also a target of many research funders as well as various governments [4–6]. The European Union as a whole for example stipulates open access availability of funded research results until 2020 [7]. As especially research projects from science, technology and medicine (STM) disciplines are government-financed or receive funds from other organizations, their researchers are increasingly confronted with open access publishing. In recent years, publishers responded to this development with offering various options for publishing journal articles open access. Also many new publishing houses were established, specializing in this business model [8, 9].

As medical informatics belongs to these STM disciplines, the researchers should also have the possibility to publish their research in open access. Certainly there are some journals that are of particular importance for publishing in this field, predominantly those who have an Impact Factor [3, 10–12]. In this context, the project “Trans-O-MIM” (full title “Strategies, models and evaluation metrics for the goal-oriented, stepwise, sustainable and fair transformation of established subscription-based scientific journals into open-access-based journals with Methods of Information in Medicine as example”) aims to investigate possibilities for transforming existing journals into open access journals [13]. At this juncture an exploration of the current options of open access publishing and the height of associated costs is from particular importance.

The subsequent sections describe which options are currently available to authors of articles in medical informatics to publish open access and how these programs are shaped with a focus on the type of open access, the fees and the licensing models. Furthermore, the suitability of various options will be discussed from different perspectives.

## Methods

The starting point of this analysis was to find out which journals are most relevant for researchers in medical informatics. As the Impact Factor is an important factor in the decision where to publish, it was concluded that all journals that had an Impact Factor in the year 2014 should be included in the investigation. Hence the 24 journals listed in Thomson Reuters Web of Science in the subject category “Medical Informatics” were chosen [14].

In the next step, the websites of the selected journals were examined. In many cases there were two different websites per journal, as some have a website within the homepage of the publishing house and an autonomous one, for example maintained by the society behind the journal. It was checked if and which options are available to publish a journal article open access. If the reviewed journal offered options for open access publishing, a further screening of conditions was performed. Important parameters were the type of open access (for example green or gold open access, open access journals or closed-access journals with a hybrid open access option), whether there are associated costs, how the articles are licensed and other interesting or important aspects if applicable. The results were collected in an Excel sheet.

## Results and Discussion

At the time of this investigation (April and May 2016) all of the 24 examined journals provided their authors with the possibility to publish their articles in open access if desired. The open access option was usually embedded in open access programs of underlying publishing houses. The names of these programs predominantly contain the word “open” so that authors immediately know what this refers to. Springer for example denotes its open access program as “Open Choice” and Wiley as “OnlineOpen” [15, 16]. In contrast to the others Elsevier only alludes to this option by writing “Supports Open Access” [17].

### Type of Open Access Option

Green open access is possible for all journals. The authors have the possibility to deposit their articles in repositories or on their websites after a defined embargo period. Differences can be found in the version that the publishers allow to deposit and in the time span. Most publishers allow authors to post the version of the accepted manuscript after six to 24 months, for example the journal *Artificial Intelligence in Medicine*, published by Elsevier, does so with an embargo period of 12 months [18].

The investigation showed that for open access publishing of articles the hybrid model is offered in most cases. Here articles are immediately published in open access and authors usually retain the copyright. Articles are published in the printed version of a journal and open access in the online version on the journal’s website [19]. Often articles of Medline indexed journals are also sent to PubMed Central where they can also be accessed immediately. Altogether 22 of the 24 examined journals worked with the hybrid model of open access publishing as shown in Table 1.

**Table 1:** Impact Factor listed journals in the subject category “Medical Informatics”. Sorted by the type of open access and the height of the 2014 Impact Factor (descending)

Medical informatics journals	Impact Factor 2014
Hybrid journals	
Statistical Methods in Medical Research	4.472
Journal of the American Medical Informatics Association	3.504
Medical Decision Making	3.240
IEEE Transactions on Information Technology in Biomedicine	2.493
Methods of Information in Medicine	2.248
Journal of Medical Systems	2.213
Journal of Biomedical Informatics	2.126
Artificial Intelligence in Medicine	2.019
International Journal of Medical Informatics	2.004
Computer Methods and Programs in Biomedicine	1.897
Statistics in Medicine	1.825
Medical & Biological Engineering & Computing	1.726
Applied Clinical Informatics	1.610
Biomedical Engineering-Biomedizinische Technik	1.458
IEEE Journal of Biomedical and Health Informatics	1.440
International Journal of Technology Assessment in Health Care	1.308
Health Information Management Journal	1.154
Journal of Evaluation in Clinical Practice	1.084
CIN-Computers Informatics Nursing	0.758
Informatics for Health & Social Care	0.735
Health Informatics Journal	0.565
Therapeutic Innovation & Regulatory Science	0.456
Open access journals	
Journal of Medical Internet Research	3.428
BMC Medical Informatics and Decision Making	1.830

The two other journals are purely open access journals offering their authors the possibility of gold open access publishing. The articles are solely published online, either in HTML or as PDF for download. It is also conspicuous that these two journals are published by open access publishing houses that only work with this business model. In contrast to the other journals their online articles are more often enriched with new technology and applications such as for example embedded metrics and social media options by trend [20, 21].

### Article Publication Charges

It is a common business model that journals charge authors for publishing their articles in open access. Though there are also other ways of financing open access, all of the big commercial publishers demand such fees. If a scientific society is involved in a journal, another financial model may be applied. In the past few years many new open access publishing houses were founded that also charge their authors or operate with different business models as for example with the above mentioned society subsidies or with a low cost infrastructure and volunteer support [22]. In the case of the examined medical informatics journals all request so-called article publication charges (APC), often also referred to as article processing charges, for publishing articles open access. The highest APC was found for the *Journal of the American Medical Informatics Association*, charging their authors 2.844 €^1^ per article. In comparison, the lowest APC was charged by the journal *Medical Decision Making* with 1.479 €. Both journals are listed under the top five pertaining to the Impact Factor of this subject category. Table 2 shows the distribution of the APCs. It can be seen that most journals demand fees in the range of 2.500–2.700 €, namely nine out of 24. Another six request fees between 2.100–2.300 € for open access publishing. The APCs of most of the others are below this amount, though not by much. The average APC of the examined journals was 2.248 €, their median fee amounts to 2.207 €.

**Table 2:** Distribution of article publication charges of Impact Factor listed journals in the field of medical informatics

Height of APCs	Number of journals	Percentage of journals
up to € 1.500	1	4%
€ 1.501 - € 1.700	2	8%
€ 1.701 - € 1.900	2	8%
€ 1.901 - € 2.100	3	13%
€ 2.101 - € 2.300	6	25%
€ 2.301 - € 2.500	0	0%
€ 2.501 - € 2.700	9	38%
€ 2.701 - € 2.900	1	4%
Average height of APCs		€ 2.248
Median height of APCs		€ 2.207

A comparison of the hybrid journals with the two pure open access journals reveals that there is no real price difference. Both charge in about the same amount as nearly half of the hybrid journals (*BMC Medical Informatics and Decision Making* 1.745 € and *Journal of Medical Internet Research* 2.209 €).

A difference between hybrid and open access journals is the further pricing. For open access journals, authors only have to pay the APCs and in some cases a low submission fee. For hybrid journals they are moreover charged for the normal publication process of printed articles. In addition to the APC, there may be for example page charges or a fee for color illustrations.

In some cases, there are different ways of getting a discount on the APCs. The journals or rather the publishers offer various options to lower the costs of open access publishing. If authors work as reviewers of a journal this might for example entitle them to a reduced fee [23]. Other journals or their publishers, Oxford University Press and JMIR amongst others, have special membership programs with advantages such as discounted APCs for their participants [24, 25].

Furthermore, some medical informatics journals have special solutions for authors from developing countries who are not able to pay the APCs. They have a waiver policy exempting eligible authors from the fees. Often this policy is based on the income levels of the World Bank ranking. Depending on from which country the corresponding author is, they may pay a lower APC or even none at all [26].

### Licensing

When publishing a research article in a journal, the publisher traditionally holds the copyright. One important aspect of open access publishing is that in this case the author retains the copyright of the article. It has become common practice that for the purpose of licensing so-called “Creative Commons” (CC) are used. They regulate how someone else can proceed with an article, for example what others are allowed to do with it (copying, distributing, modifying, for commercial or non-commercial aims) [27]. The examination of journal websites showed that most of them make use of the Creative Commons licenses. Only the two journals of Schattauer Publishers worked with copyright transfer of open access articles. But they also changed their practice within this year, and when reviewing new open access articles it can be seen that they have now also implemented Creative Commons.

However, there are differences in the licensing strategy of medical informatics journals. There are four different CC licensing types which can be combined in various ways. The most liberal type is CC-BY where credit must only be given to the authors. This type is often required by funding organizations so authors often need to use this license to meet their funders’ obligations [28]. In many cases, publishers only agree on CC-BY when authors can outline that it is required by funders.

Most publishers of the surveyed journals prefer the license CC-BY-NC or even CC-BY-NC-ND. The former means that in addition to the credit for authors the article must not be used for commercial purposes (NC). The latter furthermore prohibits the distribution of any derivatives (ND), so the article cannot be used in modified forms. Overall CC-BY-NC-ND is the most widely spread license type. Only in five cases CC-BY is offered as the standard license, for example in case of the two open access journals.

In many cases authors can decide which license type they would like to have for their article, and the publisher charges a different amount of article publication charges subject to the chosen CC license. This method is for example practiced by the *Journal of the American Medical Informatics Association*. For an article with the CC-BY-NC-ND license 2.437 € are charged and if the CC-BY license is desired the author has to pay an approximately 17% higher fee, namely an APC of 2.844 € [24].

### The Suitability of Various Open Access Options

#### Green Open Access

For authors green open access is a good possibility to publish their articles online, for example on their own website or in a repository, besides the publication in the normal subscription journal. This means that everyone can access their articles independently from a journal subscription what may lead to a higher visibility. But authors must comply with the embargo period of publishers and deposit, i.e. upload, the permitted article versions on their own. In addition, green open access often is not sufficient to comply with the regulations of funding organizations.

Readers can access green open access articles without any restrictions or limitations on access. Though they will not find newest research results in green open access due to the above mentioned embargo periods. Also, they may not receive the final version of the published article but a version of record, according to the regulations of various publishers. This version must not be distributed, modified or used for own purposes.

Publishers are not that much involved in green open access. This open access version does not mean a lot of extra work or an in- or decrease in earnings. In general publishers only have to define the frame conditions in advance. As they are the copyright holders of the articles they can determine which article version may be used and how long the authors have to wait with the deposit.

#### Hybrid Open Access

The hybrid open access option means for authors that their articles will be published within the subscription journals and additionally as an open access article online on the website of the publishers. Authors will have to transfer the copyright to the publishers and frequently the articles receive a very strict CC license in the online version. This option often results in high costs for authors. Depending on the publisher they have to pay page and color figure charges for the printed version and additional APCs for making the articles open access in the online version. The choice of the hybrid version is often also possible later on, after the publication of the article in a subscription journal. Funding organizations are sometimes critical pertaining to hybrid open access so that authors should check their regulations in advance.

Readers can easily access hybrid open access articles, as they can do with green or gold open access articles, subscriptions or payments for accessing articles are not necessary. But as the articles are in many cases under a strict CC license (e.g. CC-BY-NC or CC-BY-NC-ND), readers can only freely read them. The modification and use of the articles is not allowed if the publisher does not apply a CC-BY license. The chance of hybrid open access for readers is that they can access some articles of journals that are normally behind a paywall.

For publishers hybrid open access does mean extra work. They have to handle the manuscript as they normally do for printed articles and additionally it must be edited for the open access online version, which requires new workflows. Furthermore, there are more administrative tasks for publishers. For this extra work publishers charge the authors APCs for the hybrid open access option. They also have to implement new contracts with libraries, so-called “offsetting deals”, to combine subscription and open access publications for involved institutions.

#### Gold Open Access

When authors want to publish their research results in open access, they have the choice of various pure gold open access journals. If they decide to publish in such a journal they can normally retain their copyright as the article will be under a CC license. Some publishers may require the transfer of a non-exclusive copyright for the purpose of publishing and promoting the article. In most cases authors will be confronted with paying an APC, which can be a crucial factor in their decision where to publish. In return if they have to meet open access regulations of a funding body this option will usually be the most suitable.

For readers gold open access has many advantages. They can access gold open access articles comparatively easily, without barriers like for example paywalls. This is especially very important for readers who do not have the possibility to access normally published articles from an institution or library that has a subscription for that journal, which is often the case for researchers from developing countries. Moreover, gold open access publications are also open for interested readers from the general public. Depending on the applied CC license, which is oftentimes the most liberal CC-BY, readers can even make further use of the articles, e.g. distribute, modify and use them.

Gold open access means a relatively new publication and business model to publishers. They have to define and implement new workflows. This results mainly in a higher administration effort, for example in terms of licensing. Gold open access also means for publishers that they do not have to print these articles, which may lead to declining earnings from subscriptions or advert bookings. For compensating this loss many publishers charge APCs for gold open access articles, as described in Article Publication Charges section.

### Miscellaneous Issues

An important and often discussed issue when publishing open access in the hybrid model is the so-called “double dipping”. Journals earn on subscription fees and in addition on article publication charges, as described above in Hybrid Open Access section. To prevent this, many of the investigated journals, for example those published by SAGE and Taylor & Francis, are working with an offsetting discount. If the portion of open access articles exceeds 5%, the subscription fees are adjusted (i.e. reduced) accordingly in the following year [29].

It is also remarkable how and where open access articles can be found on the websites. This examination has shown that this differs considerably. When authors pay for open access publishing they expect that their articles can be found easily. Many journals have a separate section where all open access articles are listed. But there are also cases where no special reference is contained and these articles are simply listed amongst the traditionally published articles.

## Conclusions

Medical informatics is a research intensive discipline and research projects are often supported by funding organizations. For their research results authors have the choice to publish, among others, in many renowned journals, all offering an open access option. However, one result of this investigation is that it is often quite hard to find information on open access within the websites of the journals. In most cases a search across various linked sites is necessary to gain all information needed.

By mainly working with the hybrid model, journals are charging article publication charges to serve the interest of some authors or funders in publishing research results open access. There is an increasing demand for this option, though it is still a small percentage of all articles. This also mirrors the general situation across all disciplines. This investigation has shown that the open access option is not yet completely implemented in the daily publishing business, visible in the disparate organization for example in terms of financing or licensing.

If pure gold open access publishing in the field of medical informatics is desired, there is only the choice between two journals with an Impact Factor. As most funding bodies also support the publication in a hybrid journal so far, researchers can chose from a broad range of learned journals. Those who want to support the expansion of gold open access journals in their field can also consider publishing their articles in one of more than 20 medical informatics journals listed in the “Directory of Open Access Journals”. These journals are proven to fulfil defined criteria for gold open access, and authors can easily see if there are article publication charges including their height and which license types are offered [30].

Altogether there are many options available to publish open access. Medical informatics researchers can decide if their publications should be released traditionally or openly. Obstacles for open access are mainly the uncertainty of many authors regarding this possibility, the financial factor and the often discovered passivity of journals in promoting this option.

## Footnotes

1 APCs that were not indicated in Euro were converted; exchange rate as of May 17, 2016.
